# Evaluation of THUNDERBEAT® in open liver resection- a single-center experience

**DOI:** 10.1186/s12893-018-0423-2

**Published:** 2018-10-16

**Authors:** Bibek Aryal, Teruo Komokata, Hiroto Yasumura, Daisaku Kamiimabeppu, Maki Inoue, Kota Yoshikawa, Mamoru Kaieda, Yutaka Imoto

**Affiliations:** 1 Department of Surgery, Kagoshima Medical Center, National Hospital Organization, 8-1 Shiroyamacho, Kagoshima-shi, Kagoshima, 892-0853 Japan; 2 Cardiovascular and Gastroenterological Surgery, Graduate School of Medical and Dental Sciences, Kagoshima University, Kagoshima, Japan

**Keywords:** THUNDERBEAT®, Liver resection, Energy device

## Abstract

**Background:**

THUNDERBEAT® (TB) (Olympus Medical Systems Corp., Tokyo, Japan) is a dynamic energy system device that simultaneously delivers ultrasonically generated frictional heat energy and electrically generated bipolar energy. TB is being routinely used in various operative procedures, however, less is known about its utility in liver resection. We, herein, report our early experience of using TB in open liver resection particularly in patients with normal or near-normal liver parenchyma.

**Methods:**

We retrospectively reviewed the clinical characteristics, and evaluated the perioperative outcome of twenty-eight patients who underwent liver resection with TB, and twenty-four patients who underwent liver resection with basic procedure in our institution. The resection type was stratified into: major hepatectomy; resection of 3 or more than 3 Couinauds segments, and minor hepatectomy; resection of less than 3 Couinauds segments.

**Results:**

Liver resection time (mean ± SD) in TB group with major hepatectomy was significantly shorter: 16.7 ± 8.8 compared to 62.8 ± 39.4 min in basic procedure group (*P* < 0.0001). Accordingly, the liver resection time (mean ± SD) in TB group with minor hepatectomy was also significantly shorter, 8.3 ± 2.9 min compared to 45.2 ± 23.9 min in liver resection with basic procedure (*P* < 0.001). No significant difference was observed between the groups in terms of intraoperative blood transfusion ratio, postoperative complication and postoperative liver dysfunction.

**Conclusion:**

TB as a new energy device can offer a safe, reliable and substantially rapid liver resection particularly in patients with normal or near-normal liver parenchyma.

## Background

The steady progress achieved in the outcomes of liver resection has emerged as a result of the upgraded surgical techniques that carry the advantage of technological advancements. A majority of liver resections performed these days involves the use of some energy device for cutting, coagulation or sealing, such as Cavitron Ultrasonic Surgical Aspirator (CUSA, Tyco Healthcare, Mansfield, MA, USA), the Harmonic Scalpel (Ethicon Endo-Surgery, Cincinnati, OH, USA), the Ligasure Vessel Sealing System (Covidien, Mansfield, MA, USA) and several other dissecting sealers [1–8].

Use of energy devices in the liver parenchyma transection is popular for faster and safer hepatectomy [6, 7]. THUNDERBEAT® (TB) (Olympus Medical Systems Corp., Tokyo, Japan) is the first device to simultaneously integrate ultrasonically generated frictional heat energy and electrically generated bipolar energy [9]. The ultrasonic technology is used for rapid cutting and precise dissection while the bipolar technology performs reliable vessel sealing. TB is considered vibrant due to its ability to gain a rapid surge in temperature with minimal thermal spreading [9].

Despite plenty of reports on the utility of other devices, little is known about the relevance of TB in liver resection. In this retrospective cohort study, we used TB as a single device, assisted by Pringle maneuver with or without infra-hepatic inferior vena cave (IVC) clamping, in open liver resection, and compared the perioperative outcomes with the patients who underwent liver resection with basic procedure.

## Methods

Between April 2016 and April 2018, patients who underwent open liver resection with TB were retrospectively enrolled in the study. Similarly, a control group of patients who underwent open liver resection with basic procedure between June 2013 and February 2018 was included.

All the patients underwent routine blood tests, tumor markers evaluation, triphasic liver dynamic computed tomography (CT), and double contrast magnetic resonance imaging (MRI). Liver function was estimated using technetium-99 m galactosyl serum albumin scintigraphy, and remnant liver volume was assessed by a three-dimensional volume analyzer (SYNAPSE VINCENT; FUJIFILM Medical Co., Tokyo, Japan) in major hepatectomy.

The fibrosis score was assessed based on a five-point scale: F0 = no fibrosis, F1 = portal fibrosis without septa, F2 = few septa, F3 = numerous septa without cirrhosis, F4 = cirrhosis. Liver parenchyma with F0 was defined as normal and F1 was defined as having near normal parenchyma.

### Operative technique

Following exploratory laparotomy, with the aid of intraoperative ultrasonography, the extent of the disease along with its relationship to vascular structures was assessed in all resections. The description of the procedure in each resection group is listed below.

#### TB resection

**Fig. 1:**
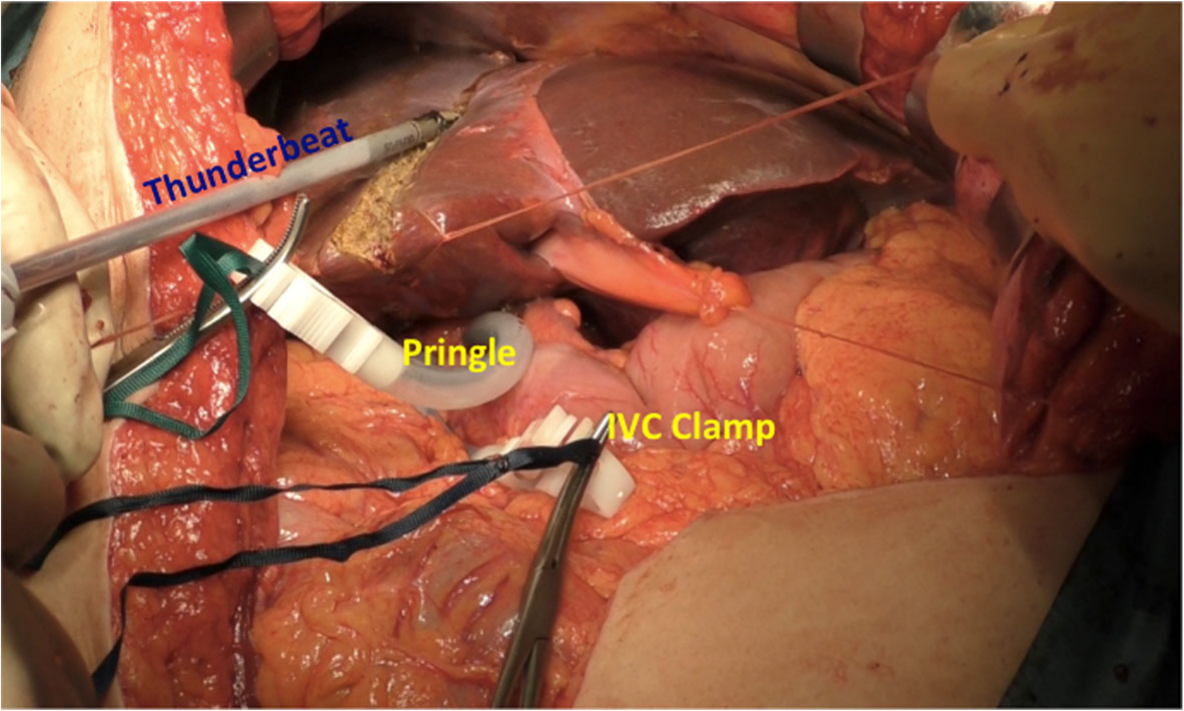
Demonstration of technique of parenchymal transection with TB Liver parenchyma being divided using TB in a patient with left hepatectomy

The liver was mobilized, and the hepatic pedicles were tapped with or without the intra-hepatic IVC taping. 1–0 Vicryl® stay sutures were placed on the hepatic edges for symmetric traction (for major hepatectomy), and the transection of the liver parenchyma was started with TB (Fig. 1). During parenchymal transection, only the pedicles equal or larger than 3rd branches of glissonian sheath or main branches of major hepatic veins were ligated and divided, the other vessels were sealed with TB. Liver resection was performed with Pringle maneuver in cycles of clamp/unclamp time of 15/5 min.

#### Basic resection

Inflow occlusion was performed by hepatic pedicle clamping along with ligation of glissonian or selective ligation of the portal vasculature and hepatic artery supplying the portion of the liver being resected. Liver was mobilized and stay sutures were placed on the hepatic edges, and about 1 cm of the superficial liver parenchyma was transected with an energy device like harmonic scalpel. Parenchymal transection was performed either by the crush-clamp method or using the CUSA. CUSA was mostly used in association with Kelly clamp rather than a single device. The exposed vessels or ducts were divided using sutures ligation. Intermittent inflow occlusion with the Pringle maneuver was performed in a similar way to the TB resection.

In both techniques, after removal of the specimen, hemostasis was achieved with Z-shaped sutures (4–0/5–0 Prolene®) when required, and Tachosil® was applied to the raw surface. A closed suction drain was placed at the end of the procedure.

### Perioperative assessment

Liver resection time indicates the duration recorded from the beginning of the parenchyma resection to the removal of the en-bloc specimen of liver.

Liver resections were defined according to the International Hepato-Pancreato-Biliary Association, Brisbane (IHPBA) 2000 nomenclature; major hepatectomy: resection of 3 or more than 3 Couinauds segments, minor hepatectomy; resection of less than 3 Couinauds segments. Clavien-Dindo classification was applied to define postoperative complication [10]. The postoperative liver dysfunction was assessed on the basis of Balzan 50–50 criteria [11].

### Statistical analyses

Statistical analyses were conducted with SPSS software (version 25; SPSS, Inc., Chicago, IL) and Graph Pad Prism (version 6.0d for Mac OS X, USA, GraphPad Software, San Diego California, USA), and were based on nonparametric tests (Mann-Whitney’s U test). The Chi-square or Fisher’s exact test was used to evaluate frequencies between categorical variables.

## Results

The clinical details and perioperative outcomes of the groups are compared in Table _***1***_. Most of the patients were diagnosed with HCC, followed by metastatic colorectal cancer (mCRC), perihilar or intra-hepatic cholangiocellular carcinoma (CCC), gallbladder cancer (GB Ca) and a few other lesions (hemangioma, IgG4-related cholangitis, cystic adenoma, hepatolithiasis and gastric cancer metastasis). On examining the fibrosis score, except HCC patients, all other patients had normal to near-normal liver parenchyma (F0 - F1). Two patients in TB group and 4 patients in basic procedure group, with mCRC, received chemotherapy after colon resection and prior to liver resection.

**Table 1:** Comparative analysis of patients’ characteristics and perioperative variables

Variables	Resection with TB (*N* = 28)	Resection with basic procedure (*N* = 24)	*P*-value
Age (Years)			
Median (Range)	73.5 (57–88)	71.0 (31–88)	0.61
Sex (M/F)			
Male, n	12	20	0.003
Female, n	16	4	
Diagnosis, n (%)			
HCC	12 (42.9)	8 (33.3)	0.687
mCRC	6 (21.4)	5 (20.8)	
CCC	4 (14.2)	2 (8.3)	
GB Ca	3 (10.7)	3 (12.5)	
Others	3 (10.7)	6 (25.0)	
Fibrosis score (HCC)			
F0 – F1, n	9	7	1.00
F2 – F4, n	3	1	
Chemotherapy prior liver resection, n	2	4	0.39
Transection time (Minutes)			
Major (mean ± SD)	16.7 ± 8.8	62.8 ± 39.4	< 0.001
Range (Min- Max)	7.0–45.0	18.0–115.0	
Transection time (Minutes)			
Minor (mean ± SD)	8.3 ± 2.9	45.2 ± 23.9	< 0.0001
Range (Min- Max)	5.5–16.0	9.0–95.0	
Intraoperative blood transfusion, n (%)	8 (28.6)	11 (45.8)	0.19
Vascular clamping			
Pringle, n	17	23	0.003
Pringle + IVC, n	11	1	
Postoperative complication			
CD ≥ 3a, n (%)	3 (7.1)	2 (8.3)	1.00
Postoperative liver dysfunction, n	0	0	NA

*M* male, *F* female, *N* number, *HCC* hepatocellular carcinoma, *mCRC* metastatic colorectal carcinoma, *CCC* cholangiocellular carcinoma, *GB Ca* gall bladder carcinoma, *SD* standard deviation, *IVC* inferior vena cava, *CD* Clavien- Dindo *NA* not applicable

Liver resection time in TB group with major hepatectomy was significantly shorter than the patients who underwent liver resection with basic procedure (16.7 ± 8.83 vs. 62.8 ± 39.4 min; *P* < 0.0001). Similarly, the minor group who underwent liver resection with TB had also significantly shorter liver resection time than the group with basically (8.4 ± 2.9 vs. 45.2 ± 23.9 min; *P* < 0.001). There was no significant difference in terms of intraoperative blood transfusion ratio between the groups. There was no difference in terms of postoperative complication between the groups; CD3a was encountered in 2 patients in TB (1 bile leakage and 1 hepatico-jejunostomy failure) and 1 patient in basic procedure group (wound infection), and CD3b was encountered in 1 patient in TB (postoperative bleeding) and 1 patient in basic procedure group (wound dehiscence). Pringle along with IVC clamping was carried out in 11 patients in TB group, and only 1 patient in the basic procedure group. None of the patients in any group developed postoperative liver dysfunction. None of the cases encountered thermal injury or device-related complications in TB group. Tumor-free margin (R0) was achieved in all cases except for one case in TB group. No mortality was recorded in any group.

## Discussion

Our data provide early evidence on the feasibility of TB as an advance device in open liver resection for selected cases. In this study, we reserved the use of TB in patients, particularly with normal to near-normal liver parenchyma, undergoing open liver resection. The commendable feature of TB observed in our study was an extensively rapid parenchyma transection time.

We have been using TB in open major or minor hepatectomy cases with HCC, perihilar CCC, T2 GB Ca, metastatic lesions and specifically in patients with normal or near-normal liver parenchyma. Parenchyma transection time was remarkably shorter even in major hepatectomies. In most of the cases, liver resection was completed with a single Pringle maneuver with or without infra-hepatic IVC clamping.

TB produces high heat compared to other energy devices (more than 200 °C compared to 100 °C for electrosurgery), thus, may not be suitable in cirrhotic, friable or steatotic liver parenchyma where the transection becomes riskier posed by the frequent distortion or displacement of intra-hepatic vessels by the tumor.

There is still inadequate evidence for one energy device to be considered superior to the other. TB has been frequently compared with other common energy devices [9, 12–17]. TB was found superior to other energy devices in animal models [15]; however, none of the existing human studies have concluded its salient advantage over the other energy devices. In a randomized controlled trial in patients undergoing laparoscopic radical hysterectomy, TB was linked to shortened operation time and less postoperative pain [18]. Use of TB in laparoscopic colorectal surgery has also concluded the safety of the device [16, 17]. There is still a paucity of evidence on its utility in liver resection. In a recent retrospective study, Badawy et al. compared TB and ultrasonic Harmonic devices in 80 patients who underwent laparoscopic liver resection and concluded that TB is safe and effective, but not superior to ultrasonic harmonic devices [16, 17]. In this study, we compared the advantages of the TB assisted liver resection with basic procedure, our results indicate that compared to the basic procedure, TB enables exceptionally rapid parenchyma transection time with no substantial difference in complications or the transfusion ratio. In this study, the number of infra-hepatic IVC clamping was higher in the TB group that is attributed to the fact that the majority of patients in the basic resection underwent minor resection.

From the prospect of cost containment, TB retains the cost-effective benefit, in that it equips the surgeon with one instrument, without the aid of any other devices, capable of any tissue dissection and sealing efficiently. TB did not only make the dissection rapid but also offered additional benefits of reliable sealing without jeopardizing the safety and oncological clearance.

Each device used in liver resection has relative advantages and disadvantages; understanding the utility of the devices are essential in deciding which energy source better suits for a specific procedure [19]. Open liver resection with TB is our preliminary experience; we, therefore, do not stipulate a clear consensus for the procedure. Furthermore, this study has limitations including a relatively small number of patients, the retrospective design of the study and limited to the open procedure. Clamp-crushing or CUSA-based technique in liver resection is conventional, standard and still commonly used [6]; we also adopt the same technique during anatomical resections. In our cohort, we encountered a few postoperative complications including a case of bile leakage in TB group; this issue also requires careful consideration in future studies. In our experience, TB appears as an appealing device in liver transection for its rapidity, safety, and simplicity; however, the clinical benefits and the inclusion criteria for liver resection need to be better clarified from a larger randomized trial.

## Conclusions

The specific use of TB in liver resection remains unclear, with little evidence available in literature.

Besides safety, the principal feature in using this device, and what endorses it peculiarly in liver resection, would be the combination of rapidity and simplicity it offers during the parenchyma transection.
